# Widespread white matter aberration is associated with the severity of apathy in amnestic Mild Cognitive Impairment: Tract-based spatial statistics analysis

**DOI:** 10.1016/j.nicl.2021.102567

**Published:** 2021-01-19

**Authors:** Tania M. Setiadi, Sander Martens, Esther M. Opmeer, Jan-Bernard C. Marsman, Shankar Tumati, Fransje E. Reesink, Peter P. De Deyn, André Aleman, Branislava Ćurčić-Blake

**Affiliations:** a Cognitive Neuroscience Center, Department of Biomedical Sciences of Cells & Systems, University of Groningen, University Medical Center Groningen, Groningen, The Netherlands; b Department of Health and Welfare, Windesheim University of Applied Science, Zwolle, The Netherlands; c Sunnybrook Research Institute and University of Toronto, Toronto, ON, Canada; d Department of Neurology, Alzheimer Center Groningen, University of Groningen, University Medical Center Groningen, Groningen, The Netherlands; e Laboratory of Neurochemistry and Behavior, Institute Born-Bunge, University of Antwerp, Antwerp, Belgium; f Department of Psychology, University of Groningen, University Medical Center Groningen, Groningen, The Netherlands

**Keywords:** ACC, Anterior cingulate cortex, ACR, Anterior corona radiata, AD, Alzheimer’s Disease, AES, Apathy evaluation scale, aMCI, amnestic Mild Cognitive Impairment, ATR, Anterior thalamic radiation, BNT, Boston naming test, CC, Corpus callosum, CST, Corticospinal tract, DTI, Diffusion tensor imaging, DS, Digit Span, FA, Fractional anisotropy, FSL, Functional MRI of the Brain (FMRIB) Software Library, GDS, Geriatric depression scale, IFOF, Inferior fronto-occipital fasciculus, LPC, Lateral parietal cortex, MMSE, Mini mental state examination, NPS, Neuropsychiatric symptoms, OFC, Orbitofrontal cortex, RAVLT, Rey auditory verbal learning test, SCR, Superior corona radiata, SDMT, Symbol digit modalities test, SLF, Superior longitudinal fasciculus, TBSS, Tract-based spatial statistics, TFCE, Threshold-free cluster enhancement, TMT, Trail making test, UF, Uncinate fasciculus, VS, Ventral striatum, VTA, Ventral tegmental area, Apathy, amnestic Mild Cognitive Impairment, Goal-directed behavior, Diffusion tensor imaging, Tract-based spatial statistics

## Abstract

Apathy is recognized as a prevalent behavioral symptom of amnestic Mild Cognitive Impairment (aMCI). In aMCI, apathy is associated with an increased risk and increases the risk of progression to Alzheimer’s Disease (AD). Previous DTI study in aMCI showed that apathy has been associated with white matter alterations in the cingulum, middle and inferior longitudinal fasciculus, fornix, and uncinate fasciculus. However, the underlying white matter correlates associated with apathy in aMCI are still unclear. We investigated this relationship using whole-brain diffusion tensor imaging (DTI). Twenty-nine aMCI patients and 20 matched cognitively healthy controls were included. Apathy severity was assessed using the Apathy Evaluation Scale Clinician version. We applied the tract-based spatial statistics analyses to DTI parameters: fractional anisotropy (FA), mean diffusivity, axial diffusivity, and radial diffusivity to investigate changes in white matter pathways associated with the severity of apathy. No significant difference was found in any of the DTI parameters between aMCI and the control group. In aMCI, higher severity of apathy was associated with lower FA in various white matter pathways including the left anterior part of inferior fronto-occipital fasciculus/uncinate fasciculus, genu and body of the corpus callosum, superior and anterior corona radiata, anterior thalamic radiation of both hemispheres and in the right superior longitudinal fasciculus/anterior segment of arcuate fasciculus (*p* < .05, TFCE-corrected) after controlling for age, gender and GDS non-apathy. A trend association was observed in the right posterior corona radiata and corticospinal tract/internal capsule, and bilateral forceps minor (*p* < .065, TFCE-corrected). In conclusion, in aMCI, severity of apathy is associated with aberrant white matter integrity in widely distributed pathways, within and between hemispheres.

## Highlights

• In aMCI, apathy severity was associated with lower FA in widespread WM pathways.
• WM aberrations are related to apathy severity after controlling for depression.
• Disruptions related to apathy severity are not limited to frontal-subcortical area.

## 1 Introduction

Mild cognitive impairment (MCI) refers to a decline in cognitive functioning that goes beyond declines related to normal ageing, but it is not as severe as dementia. People with MCI do not meet the criteria for dementia, and have normal activities of daily living. Amnestic MCI (aMCI) is a subtype of MCI mainly marked by impairment in episodic memory, but can co-occur with deficits in other cognitive domains (Petersen, 2004). In people with MCI, cognitive decline is often accompanied by neuropsychiatric symptoms (NPS), of which apathy (varying from 3.1% to 50.5%) and depression (32%) are the most prevalent (Fernandez-Martinez et al., 2010, Geda et al., 2008, Ismail et al., 2016, Lanctôt et al., 2017, Onyike et al., 2007, Sherman et al., 2018). Other symptoms include agitation and disorientation (prevalence 4–35%) and delusions and hallucinations (3–14%) (Gallagher et al., 2017). Apathy and depression often co-occur with one another and although they are distinct constructs, their symptoms may overlap (Marin et al., 1993, Tagariello et al., 2009). Apathy occurs in several psychiatric and neurological disorders [e.g. Marin, 1990], its presence in aMCI, independent of depression, is related to a greater risk of progression to AD (Chilovi et al., 2009, Palmer et al., 2010, Richard et al., 2012, van Dalen et al., 2018). Moreover, apathy has been associated with adverse consequences such as functional decline, physical and social inactivity, increased caregiver distress (Clarke et al., 2010), decreased quality of life (Conde-Sala et al., 2016), and higher mortality (Nijsten et al., 2017).

The definition of apathy has altered in the past decades (Chase, 2011). Apathy was primarily defined as “a lack of motivation”. It has been thought to be comprised of several factors related to cognitive, behavior and emotion (Marin, 1991). In 2009, an international consensus committee regarded apathy as a syndrome of diminished motivation that is persistent over time (Robert et al., 2009). It included symptoms in at least two out of three dimensions, representing behavioral, cognitive, and emotional apathy, concordant with the original concept of apathy as a ‘multidimensional’ entity described by Marin (1991). Another definition considers apathy to be “a reduction in goal-directed behavior” (Levy and Dubois, 2006). Levy and Dubois (2006) proposed three components of apathy (i.e. cognitive, emotional-affective and auto-activation) similar to Marin’s definition, except for the auto-activation, which represents problems with initiation of behaviors and cognition.

Levy and Dubois proposed an apathy model based on the impairment of distinct prefrontal-basal ganglia circuits (Levy and Dubois, 2006, Levy, 2012). They suggested that lesions of the orbito-medial prefrontal cortex and basal ganglia (e.g. the ventral striatum) are associated with apathy through difficulties in providing emotional value to a given behavioral context (disruption of “emotional-affective” process) (Hollerman et al., 1998). The orbito-medial PFC is connected to limbic region via the uncinate fasciculus. It is considered that the input from the limbic to the orbito-medial PFC influences ongoing behavior (Öngür and Price, 2000). Lesions of the frontal lobes (the dorsal prefrontal cortex) and basal ganglia (e.g., the dorsal caudate) are believed to contribute to apathy through diminished ability to generate plans required to successfully complete an action (disruption of the associative/“cognitive” process that is related to difficulties in elaborating new patterns of behavior (Levy, 2012). And finally, the dlPFC is an important brain region for executive function such as planning and working memory. The lateral PFC is also closely connected with the dorsal part of the caudate nucleus, which together contribute to executive functioning (Barbey et al., 2013, Carpenter et al., 2000). Bilateral lesions in the prefrontal-basal ganglia circuits or the additional lesions in the cognitive and limbic territories may lead to “auto-activation” deficit syndrome, which refers to difficulties in initiating motor programs to complete behavior. Lesions in the basal ganglia may lead to apathy because basal ganglia processing may no longer be able to generate relevant neural signals to its output targets in the prefrontal cognitive and limbic territories (Levy and Dubois, 2006, Levy, 2012).

Reviews of the published neuroimaging literature confirm the involvement of these circuits, in addition to other brain regions. More specifically, across various neuroimaging modalities and pathologies, apathy is consistently associated with disruptions in medial frontal cortex, including the anterior cingulate cortex (ACC) and orbitofrontal cortex (OFC), and subcortical regions such as ventral striatum (VS) (Kos et al., 2016, Le Heron et al., 2018). In AD and MCI, apathy is associated with structural alterations of gray matter in the prefrontal cortex, medial frontal cortex (i.e. anterior cingulate cortex (ACC) and orbitofrontal cortex (OFC)), subcortical areas (VS, medial thalamus, VTA), basal ganglia and parietal cortex (Kos et al., 2016, Raimo et al., 2019, Stella et al., 2014, Theleritis et al., 2014). Despite these findings, only a few studies have investigated whether any alterations in white matter tracts connecting these brain regions may also be associated with apathy.

One way to investigate white matter structure would be to use diffusion tensor imaging (DTI). DTI is a quantitative and non-invasive MRI technique that provides quantitative information regarding the diffusion of water molecules. Fractional anisotropy (FA) and mean diffusivity (MD) are derived using DTI and reflect the directionality of water diffusion within the tract and the magnitude of overall water diffusion, respectively (Basser et al., 1994a, Le Bihan, 2003, Tournier et al., 2011). DTI has been used to investigate white matter structural alterations in the brain and has shown to be useful in identifying changes even in the early stage of AD (Zhang et al., 2014). Previous DTI studies in AD have demonstrated the association between apathy and reduced FA in the left anterior cingulum (Hahn et al., 2013, Kim et al., 2011, Tighe et al., 2012), the white matter (WM) underlying bilateral parietal cortex, right anterior cingulate cortex, and thalamus (Ota et al., 2012), the corpus callosum, right superior longitudinal fasciculus (SLF), and bilateral uncinate fasciculus (UF) (Hahn et al., 2013). However, in aMCI, the association between apathy severity and the white matter changes is still unclear. To date there has only been a single study investigating the WM correlates of apathy in aMCI patients. In a DTI study of 20 aMCI patients using voxel-based analysis, Cacciari and colleagues (2010) reported a significant association between apathy and increased mean diffusivity in the right temporal lobe (more specifically the uncinate fasciculus [UF], middle longitudinal fasciculus [MLF], and inferior longitudinal fasciculus [ILF]), in the parathalamic WM, fornix and posterior cingulum, as well as decreased FA in similar WM areas except for the MLF. Voxel-based analysis, however, has several limitations such as being prone to registration errors (Ashburner and Friston, 2000) and when applied to FA data, the random selection of smoothing factors might also affect the final results (Smith et al., 2006). To improve these issues, we conducted a tract-based spatial statistics (TBSS) analysis, a hypothesis-free technique that detects structural changes throughout the whole brain employing a “skeletonization” step to mitigate image misalignments and prevents the need for data smoothing (Smith et al., 2006).

Several above-mentioned brain structures are associated with goal-directed behavior and motivation, such as the ACC, VS, OFC, dlPFC (Le Heron et al., 2018). The circuitry between these regions and striatum and thalamus has also been implicated in drive, planning and cognition for the development and expression of goal-directed behaviors (Haber and Calzavara, 2009). We discussed above the aspects of motivation and goal-directed behavior for apathy. Therefore, we hypothesize that aberration in this circuitry will be associated with apathy. We also explored any other possible involvement of white matter tracts in apathy using TBSS, while controlling for depressive symptoms.

## 2 Material and methods

### 2.1 Subjects

Thirty-one amnestic MCI patients (aMCI group) and 20 cognitively healthy controls (control group) were included in this study. Two aMCI subjects were excluded from the analysis because one subject did not have diffusion scans and the diffusion weighted scan of the second subject was corrupted. Therefore, the data of twenty-nine aMCI subjects were included in the final analysis. The study was approved by the medical ethical review board of the University Medical Center Groningen and all subjects had given their written informed consent prior to the study in accordance with the Declaration of Helsinki. Diagnoses of aMCI and clinical apathy were made based on a neuropsychological evaluation by a trained neuropsychologist and further confirmed by a neurologist. aMCI diagnosis was made according to the criteria of Petersen (Petersen et al., 1999) and includes: (1) memory complaints by the patient or observed by a close other; (2) a score lower than 1.5 standard deviations (SDs) below the normative control values on a memory test; (3) a score of ≥24 on the Mini Mental State Examination (MMSE); (4) normal activities of daily living; (5) no symptoms of dementia (based on clinical examination by a neurologist and interview with a knowledgeable informant). Apathy was diagnosed clinically in eight subjects with aMCI based on the clinical diagnostic criteria by Robert et al., 2009, Robert et al., 2018 which comprised of: (1) diminished motivation that must be present for at least 4 weeks; (2) presence of two of the three dimensions of apathy (reduced goal-directed behavior, goal-directed cognitive activity, and emotions); (3) there are identifiable functional impairments attributable to apathy; (4) exclusion criteria are specified to exclude symptoms and conditions mimicking apathy. Apathy severity was determined with the Apathy Evaluation Scale Clinician version (AES-C; see Section 2.1.1. below). Cognitively healthy participants in the control group were recruited through advertisements. The control group was matched with the aMCI group for age, gender, and education. Inclusion criteria for the control group were a MMSE score of ≥27, no subjective or objective memory complaints, and normal performance on the neuropsychological tests. General exclusion criteria included the use of medication that may affect cognition, MRI contra-indications, any current or a history of psychiatric or neurological disorders with the exception of depressive symptoms, head trauma accompanied by a loss of consciousness, and anatomical abnormalities (e.g. brain tumor) found in the MRI scan.

#### 2.1.1 Behavioral and neuropsychological assessment

All subjects underwent behavioral and neuropsychological tests. The severity of apathy was evaluated using the Apathy Evaluation Scale Clinician version (AES-C), which is comprised of 18 items scored on a 4-point Likert-type scale based on the subject’s thoughts, feelings, and actions (Clarke et al., 2007, Kazui et al., 2017, Marin et al., 1991). Total scores ranged between 18 and 72, with higher scores indicating higher levels of apathy. In addition, although AES-C is designed as a unitary measure (Clarke et al., 2007), we calculated the scores of different “components” within the AES-C: Cognitive, Behavioral, and Emotional (Marin et al., 1991). Cognitive apathy consisted of the following items no. 1, 3, 4, 5, 8, 11, 13, 16. Behavioral apathy consisted of items no. 2, 6, 9, 10, 12. Two items - No. 7 and 14 comprised emotional apathy and the three remaining items were described as others (i.e. “s/he has an accurate understanding of her/his problems”; “s/he has initiative”; “s/he has motivation”) (Marin et al., 1991). The sub-component total score was obtained by summing up all the scores on all the items under each component. The 30-item Geriatric Depression Scale (GDS) was used to assess depressive symptoms (Yesavage et al., 1982). The GDS is a self-report screening scale which consists of “yes/no” questions with higher scores indicating more depressive symptoms. In addition, we calculated a sub-score of the GDS by excluding six items which are related to apathy (Adams et al., 2004). This sub-score (i.e. GDS non-apathy) had a range of 0–24 and was used as a covariate in the statistical analyses.

A set of neuropsychological tests was administered, including the Mini Mental State Examination (MMSE) to assess general cognitive ability (Folstein et al., 1975), the 15-word Rey Auditory Verbal Learning Test (RAVLT; also used for aMCI diagnosis) immediate and delayed recall, Digit Span (DS), Symbol Digit Modalities Test (SDMT), Stroop Test, Trail Making Test (TMT), Hayling Test, and the Boston Naming Test (BNT). These specific tests were selected because they have been well validated (Lezak et al., 2004) and together provide a reliable estimate of general cognitive ability.

#### 2.1.2 Statistical analysis of demographic data

Statistical analysis for demographic, behavioral and neuropsychological data were carried out using IBM SPSS version 25.0 (IBM Corp, Armonk, NY, 2014). Differences between groups were analyzed using the Mann-Whitney *U* test for non-normally distributed data or Student’s *T*-test for normally distributed data, and Chi-square tests for gender and handedness. Associations between apathy severity and other variables in each group were analyzed with Spearman correlations. Partial correlations between AES and MMSE and cognitive functions scores were determined after controlling for age and education. The level of significance was set at *p <* .05 (two-sided).

### 2.2 Diffusion tensor imaging (DTI)

#### 2.2.1 Magnetic resonance imaging acquisition

Diffusion-weighted images were obtained with a 3T Phillips Intera scanner (Phillips Medical Systems, Best, The Netherlands) equipped with a 32-channel synergy SENSE head coil, using a single-shot pulsed gradient spin-echo, echo-planar imaging (EPI) sequence. Restraining foams on the subject’s head and earplugs were used to minimize the head movement and to reduce scanner noise. Diffusion-weighted images were acquired along 60 isotropic gradient directions (b = 1000 s/mm^2^) and one unweighted b = 0 s/mm image together with reversed k-space with two acquisition directions: anteroposterior and posteroanterior. The acquisition parameters for 55 axial slices of 2.5 mm thickness were as follows: repetition time [TR] = 8947 ms; echo time [TE] = 60.1 ms; in-plane field of view [FOV] = 240x240 mm2; image resolution 96 × 96 mm; flip angle [FA] = 90°). The acquisition time for each scan was 10.3 min.

#### 2.2.2 Diffusion tensor MRI processing

The DTI images were processed within the Functional MRI of the Brain (FMRIB) Software Library (FSL) toolbox version 5.0 (www.fmrib.ox.ac.uk/fsl) (Smith et al., 2004). The pair of diffusion-weighted images acquired in opposite phase encoding directions were used to estimate the susceptibility-induced-off-resonance field with the “topup” tool (Andersson et al., 2003), which yielded a single corrected image. This output was passed into the “eddy” tool for eddy currents and subject movement corrections (Andersson and Sotiropoulos, 2016). All images were visually inspected before and after corrections. We then created a mask by excluding non-brain tissue from the corrected image with a b value = 0 using the “Brain Extraction Tool (BET)” inside the FSL package (Smith, 2002). The diffusion tensor model was fitted using the least squares method (Basser et al., 1994b) employed in the “DTIFIT” toolbox inside the FMRIB’s Diffusion Toolbox (FDT), which created fractional anisotropy (FA), mean diffusivity (MD) and eigenvalue maps.

#### 2.2.3 Tract-based spatial statistics (TBSS) analysis

Voxel-wise statistical analyses of the skeletonized FA data were performed using TBSS (Smith et al., 2006), which is also a part of the FSL toolbox (Smith et al., 2004). FA images of all subjects were nonlinearly registered onto the FMRIB58 FA template (http://fsl.fmrib.ox.ac.uk/fsl/fslwiki/FMRIB58_FA) as a target and aligned the images onto 1 × 1 × 1 mm Montreal Neurological Institute (MNI) 152 space*.* The transformed FA images were then averaged, resulting in a derived mean of all FA images that were used to generate a mean FA “skeleton” (i.e. the core of all fiber bundles common to all subjects). The aligned FA image of each subject was further projected onto the “mean FA skeleton”, thresholded at 0.2 to exclude GM voxels or cross-subject image misalignment. This projected-skeletonized data was then fed into the voxel-wise statistics across subjects.

To estimate the voxel-wise FA differences between aMCI and the control group, as well as the association between apathy (AES-C scores) and FA in the patient group, individual skeleton images were inputted to the general linear model (GLM) analysis. The statistical significance was estimated with permutation-based nonparametric inference (5000 permutations using FSL randomize toolbox inside TBSS (Winkler et al., 2014). For association between apathy (AES-C score) and FA, age, gender and GDS-non apathy were used as covariates. Age and gender have been demonstrated to be related with FA variance in WM (Hsu et al., 2008, Inano et al., 2011). Gender differences have also been noted in the neural correlates associated with apathy in a subclinical apathy sample (Spalletta et al., 2013). Moreover, since depressive symptoms often co-occur with apathy (Tagariello et al., 2009) and have been associated with WM (Wen et al., 2014), we also considered GDS non-apathy sub-scores as an important confounder and therefore included these scores as a covariate. All covariate data were mean-centered. To include as many subjects as possible, we firstly included all subjects (n = 29) and filled in the missing GDS non-apathy data with the average of the scores. Subsequently, to check whether adding these missing values changes the observed association, we also performed the analysis by excluding the one subject who did not have a GDS score (n = 28). To adjust for multiple comparisons across space, we used the Threshold-Free Cluster Enhancement (TFCE) with a specified significance level of *p <* .05 (Smith and Nichols, 2009). The John Hopkins University (JHU)-International Consortium of Brain Mapping DTI-81 WM label and JHU white matter tractography atlases (Mori et al., 2005, Wakana et al., 2004), and the atlas of human brain connections (Catani et al., 2013) were used to identify white matter areas.

In line with previous research suggesting that exploring all tensor behavior, rather than FA alone, may better captures the full extent of WM changes in AD (Acosta-Cabronero et al., 2010), we also implemented the same steps of TBSS analyses to other diffusion tensor parameters (i.e. mean diffusivity [MD], axial diffusivity [AD], and radial diffusivity [RD]). The sum of the second and third eigenvalues of the diffusion tensor was used to define RD.

## 3 Results

### 3.1 Subjects’ characteristics

Demographic, behavioral and neuropsychological data of the aMCI and control groups are presented in Table 1. Using the cut-off score of 37.5 (Marin et al., 1991), eight aMCI patients but none of the controls were considered as having clinical apathy. Moreover, following the general GDS-30′s cut-off score of 11 (Yesavage et al., 1982), 17 subjects with aMCI and 19 controls did not meet criteria for depression (scored 0–10), 9 aMCI and 1 control exhibited mild depressive symptoms (scored 11–20), and 2 aMCI patients were considered to have moderate to severe depressive symptoms (scored above 20). There was no difference between groups on age, gender, education level, MMSE and AES-C scores. The patient group had higher levels of GDS and GDS non-apathy (indicating depression independent of apathy) scores relative to the control group. As per group definitions, the aMCI group showed lower scores on the 15-word RAVLT immediate recall (t = −4.4, *p* < .001) and delayed recall (t = −5.0, *p* < .001), compared to the control group. There was no significant difference between the groups in executive functions, processing speed, or naming performance.

**Table 1:** Demographic and Behavioral Characteristics.

	aMCI Group (n = 29)		Control Group (n = 20)		Group comparison (*p*)
	Mean (*SD*)	Range	Mean (*SD*)	Range	
Age	67.3 (4.9)	60–78	67 (5.1)	61–79	U = 272.5 (0.72)
Gender (males)	21		13		X*^2^* = 0.31 (0.58)
Handedness (right)	26		20		*X^2^* = 2.20 (0.14)
Education^1^	5.3 (1.1)	3–7	5.7 (0.7)	4–7	U = 237.5 (0.26)
“University” level (%)^2^	41.4		60		
MMSE	28.6 (1.7)	22–30	28.8 (1.1)	27–30	U = 287 (0.95)
GDS	8.43 (7.1)	0–25	2.4 (3.6)	0–12	U = 110.5 (<0.01)*
GDS non-apathy	6.25 (5.5)	0–20	1.6 (2.6)	0–9	U = 115 (<0.01)*
AES-C	31.2 (10.7)	18–54	26.2 (4.6)	20–38	U = 223 (0.17)
RAVLT IR	31.6 (7.1)	17–46	41.5 (8.3)	26–54	t = −4.4 (<0.01)*
RAVLT DR	5.3 (2.0)	1–10	8.8 (2.7)	3–13	t = −5.0 (<0.01)*
Digit Span F	5.7 (1.2)	4–8	6.2 (1.4)	4–8	U = 231.5 (0.21)
Digit Span B	4.6 (0.8)	3–6	4.8 (1.0)	3–7	U = 277.5 (0.78)
SDMT	46.0 (7.8)	25–60	50.4 (7.7)	39–64	t = −1.90 (0.06)
TMT A	38.7 (11.8)	20–64	39.9 (17.8)	19–90	U = 269 (0.82)
TMT B	45.3 (26.7)	17–120	43.5 (32.9)	8–140	U = 225 (0.48)
BNT	26.7 (2.8)	20–30	26.5 (2.9)	20.5–30	U = 276.5 (0.78)
Stroop I	48.3 (9.4)	30–77	46.2 (7.1)	34–61	U = 241.5 (0.47)
Stroop II	63.0 (9.6)	48–87	61.4 (8.7)	49–80	U = 246.5 (0.54)
Stroop Interference	58.4 (31.9)	20.5–153.5	49.4 (14.5)	36.5–85	U = 248 (0.56)
Hayling test	4.0 (1.9)	1–7	4.7 (2.0)	1–8	U = 220 (0.20)

Group comparisons were performed with Student’s *T*-test or Mann-Whitney *U* test for continuous data, and Chi-square tests for gender and handedness. ^1^Education level was rated according to the Dutch education system (Verhage, 1964); ^2^″University” level (i.e. Verhage 6 “Finished high level of secondary education” and Verhage 7 “University level degree”). aMCI, amnestic Mild Cognitive Impairment; MMSE, Mini Mental State Examination; GDS, Geriatric Depression Scale; AES-C, Apathy Evaluation Scale Clinician version; RAVLT-IR, Rey Auditory Verbal Learning Test – Immediate Recall; RAVLT-DR, Rey Auditory Verbal Learning Test – Delayed Recall; DS, Digit Span Test; SDMT, Symbol Digit Modalities Test; TMT, Trail Making Test; BNT, Boston Naming Test. *Significant at *p* < .01 (2-tailed).

In the aMCI group, higher AES-C scores were significantly associated with higher GDS scores (r = 0.54, *p* = .003) and with the GDS non-apathy sub-scores (r = 0.42, *p* = .03). After including age and education as covariates, we found that higher AES-C was significantly associated with Stroop I (r = 0.50, *p* = .01) but not with other cognitive tests. Hence, we added the Stroop I as a covariate in the DTI analysis.

The AES-C sub-component scores did not differ between groups (Supplementary Table 1). In the aMCI group, Spearman’s correlation showed significant association between higher GDS scores and behavioral apathy sub-component (r = 0.65, *p* < .001) and emotional apathy sub-component scores (r = 0.64, *p* < .001) and between the GDS non-apathy scores and behavioral and emotional apathy (r = 0.54, *p* = .003 and r = 0.57, *p* = .002, respectively).

### 3.2 White matter integrity differences between the aMCI and control group

A voxel-wise TBSS comparison showed that aMCI patients did not significantly differ in any of the DTI parameters (FA, MD, AD or RD) compared to the control group (at a threshold of *p* < .05, TFCE-corrected).

### 3.3 Association between AES-C and DTI parameters

**Fig. 1:**
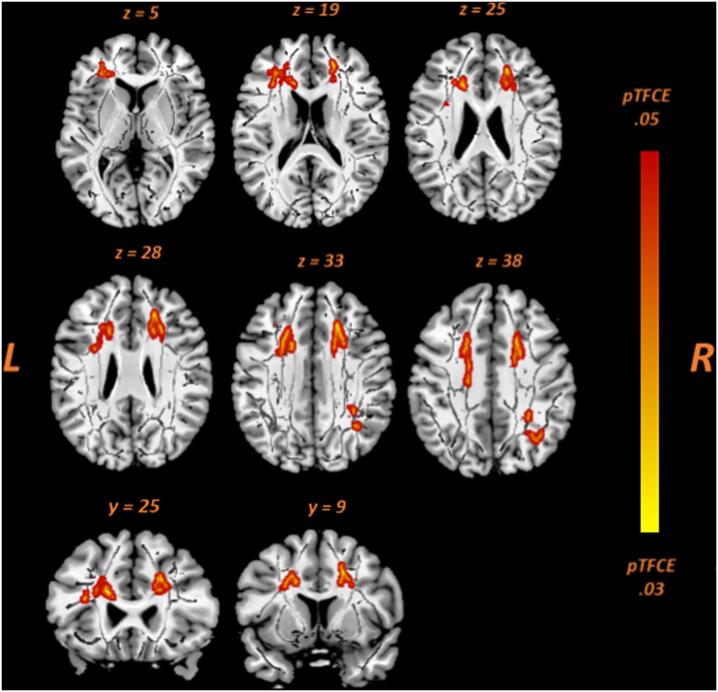
Voxel-wise correlation analyses between apathy scores (AES-C) and FA in aMCI patients (n = 29). The following are depicted (red-yellow): left IFOF/UF (z = 5); genu CC (z = 19, y = 25); body CC (z = 33, y = 9); ACR bilateral (z = 25, y = 25); SCR bilateral (z = 38); ATR bilateral (z = 19, z = 28, y = 25); right SLF (z = 33 and z = 38). For better visualization, the results were thickened using the “tbss-fill” command. (For interpretation of the references to colour in this figure legend, the reader is referred to the web version of this article.)

We initially examined the association between the severity of apathy and DTI-derived parameters (FA, MD, AD, and RD) in 29 aMCI patients while controlling for age, gender and GDS non-apathy. As shown in Fig. 1, higher AES-C scores were significantly associated with decreased FA (*p* < .05, TFCE-corrected) in the left anterior part of inferior fronto-occipital fasciculus (IFOF)/uncinate fasciculus (UF), genu and body of the corpus callosum, bilateral superior and anterior corona radiata (SCR and ACR), and bilateral anterior thalamic radiation (ATR). In the right hemisphere, inverse associations were also exhibited in the SLF/anterior segment of arcuate fasciculus (AF) (See Supplementary Table 2). Moreover, a trend association was observed between higher AES-C scores and lower FA in the right posterior corona radiata and corticospinal tract (CST)/internal capsule, and bilateral forceps minor (*p* < .065, TFCE-corrected) (Supplementary Table 3). No significant association was observed between apathy and other DTI measures such as MD, AD, or RD. In addition, since there was a significant association between AES-C and Stroop I scores in the aMCI group, indicating slower processing speed (r = 0.50, *p* = .01), we added Stroop I as a covariate in the neuroimaging analysis. After correcting for age, gender, GDS non-apathy and Stroop I, the association between apathy severity and FA was weaker (*p* = .089, TFCE-corrected).

We also analyzed the WM correlates of apathy in a combined group of aMCI and cognitively healthy controls (n = 49). In this combined group, after controlling for age, gender, and GDS-non apathy, the association between the severity of apathy and decreased FA was found in similar areas at a lower threshold (*p* < .08, TFCE-corrected) (See also Supplementary Table 2).

In the control group only (n = 20), we found no significant association between the severity of apathy and any of the DTI-derived parameters.

### 3.4 Association between AES-C sub-component and DTI parameters

There was no significant association between any of the apathy sub-component scores (i.e. cognitive apathy, behavioral apathy or emotional apathy) and DTI parameters either in the aMCI group (n = 29) or in the combined group (n = 49).

## 4 Discussion

In this study, we investigated the association between the severity of apathy and whole brain DTI measures in aMCI patients. After taking the confounding effects of age, gender, and GDS non-apathy sub-scores into account, we found a widespread white matter integrity aberration associated with apathy severity. More specifically, higher apathy severity, as indicated by higher AES-C scores, was significantly associated with lower fractional anisotropy (FA) in widespread white matter pathways including commissural (i.e. corpus callosum), association (i.e. IFOF, SLF/AF) and projection (i.e. anterior and superior corona radiata, anterior thalamic radiation) pathways. FA is the degree of directional restriction of the diffusion of water, reflecting the integrity of the underlying WM tract (Mori and Zhang, 2006). Therefore, decreased FA values indicate a change in white matter integrity which may be due to various microstructural processes such as demyelination, axonal degradation, or gliosis (Beaulieu, 2002).

We found a moderate positive correlation between AES-C scores and GDS non-apathy in the patients’ group. This is in line with the notion that apathy and depression have overlapping symptoms (Clarke et al., 2007) although they have different constructs (Starkstein et al., 2009). Therefore, we took GDS non-apathy into account in the analysis.

We did not find significant associations (after correction for multiple comparisons) between AES-C sub-components and DTI measures. Thus, our findings of widespread WM differences correlated with apathy symptoms could not explain the multidimensional construct of apathy. This is not necessarily indicative that the sub-components are not relevant to drive the changes in WM in aMCI participants, but it could mean that a larger sample of aMCI participants with better distribution of sub-scores is needed to further delineate significance of these dimensions.

Surprisingly, we observed no difference in FA values between patients and controls in the whole brain analysis, in contrast to previous studies (Liu et al., 2013, Zhuang et al., 2013). This might be due to different patient characteristics compared to the other studies. In our study, the groups differed in memory and depression, but their global cognition is relatively intact and similar. Zhuang et al. (2013) compared cognitively normal controls, ‘early’ aMCI (i.e. people who have recently converted to aMCI) and ‘late’ aMCI (i.e. people with stable diagnosis over 2 years). Indeed, FA was significantly different between controls and ‘late’ aMCI patients, but not between controls and ‘early’ aMCI patients (Zhuang et al., 2013). In our study, aMCI diagnosis was made at a single time point. Thus, the different finding might be explained by different aMCI stages, which may have various degrees of WM alterations. Liu et al. (2013) found a significant difference in global cognition scores (assessed with Montreal Cognitive Assessment) between aMCI and controls (Liu et al., 2013), meaning that their two groups of participants differed on the global cognition, while in our study MMSE scores were similar in the two groups.

In a combined group of patients and controls, we only found a trend association between aberrations in white matter integrity and higher levels of apathy in the similar WM tracts after correcting for age, gender and GDS non-apathy scores. This is in line with our findings of no significant correlation between AES-C and DTI measures in the control group. In addition, we have to take our conclusions with caution, as the significance of this results drops towards a trend with adding additional covariate Stroop I. It should be noted, though, that on the sample of 29 subjects, adding 4 covariates decreases the power substantially. Therefore, future studies should repeat this analysis with a larger sample.

### 4.1 The commissural pathways and apathy

Our findings revealed that within the aMCI group, higher levels of apathy correlated with decreased FA, mainly in the anterior part of the corpus callosum (CC), the genu and its anterior extension radiating fibers, i.e. right forceps minor and the body of the CC. Previous studies on AD (Hahn et al., 2013), Parkinson’s disease (Zhang et al., 2018) and HIV patients (Hoare et al., 2010) have also revealed an inverse relationship between apathy severity and FA in the genu and body of the corpus callosum. The CC is the largest commissural tract in the human brain that connects the left and right hemispheres and facilitates interhemispheric communication. Structurally, from anterior to posterior, the CC comprises of the genu, body and splenium. The genu connects the prefrontal and OFC of both hemispheres, while the forceps minor connects to the medial and lateral frontal lobes (Catani et al., 2013, Clark et al., 2018). The prefrontal cortex is primarily associated with executive functions, where the ventromedial PFC (vmPFC) is known to be important for emotional/motivational executive functions (i.e. coordinating cognition and emotion, emotional and social decision making) and the dorsolateral PFC (dlPFC) plays a role in cognitive aspects of executive functions (i.e. planning, response inhibition, working memory) (Ardila, 2008, Robinson et al., 2014, Stuss, 2011). Impairment of the OFC is associated with poor motivation (Massimo et al., 2015). The body fibers interconnect the motor cortices (i.e. premotor, precentral frontal cortex, and parietal lobes), controlling execution of movement, motor control, and internal generation of movement (Hofer and Frahm, 2006). Therefore, we suggest that lower FA in the genu, forceps minor, and body of the corpus callosum between the hemispheres may compromise communication and integration of motivation, cognition, and motor processing between the two hemispheres, thus leading to more severe apathy.

### 4.2 Association pathways and apathy

Our findings show that higher apathy severity is associated with changes in WM integrity in the major association tracts connecting frontal and other cortices (parietal, temporal, and occipital), e.g. in the right superior longitudinal fasciculus (SLF)/anterior segment of arcuate fasciculus (AF) and the left anterior part of the inferior fronto-occipital fasciculus (IFOF).

The SLF/AF connects the lateral frontal cortex with the dorsolateral parietal and temporal lobes around the Sylvian fissure (Catani et al., 2013). In the current study we observed lower FA particularly in the anterior segment of AF, which links the inferior parietal lobule (Geschwind’s territory) to Broca’s area in the frontal lobe. Broca’s area is involved in speech production (Catani and Ffytche, 2005, Matsumoto et al., 2004). The Geschwind’s territory is a place where sensory and motor impulses integrate (Jardri et al., 2007). The anterior segment of the AF and the ventral branch of the SLF are rarely distinguished in the literature, and they are considered to be either part of the sensory-motor SLF system or the arcuate language network (Catani et al., 2013). However, our findings showed lower FA mainly in the SLF of the right hemisphere, which is known to play a role in attention, visuospatial processing, and spatial working memory (Catani and Ffytche, 2005, De Schotten et al., 2005). Indeed, apathy has been reported to be associated with impairments in attention and working memory (Robert et al., 2006). We suggest that the aberrant connectivity between frontal and parietal cortices, especially in the right hemisphere, may disrupt the spatial attention network and affect the steps needed to achieve successful goal-directed behavior (Brown and Pluck, 2000).

We also observed disruptions in WM tract which connects the frontal and temporal (and occipital) cortices, reflected in lower FA in the left anterior part of IFOF. The IFOF connects the mediolateral orbital frontal cortex (OFC) and proceeds ventrally to posterior temporal lobe and terminates in the inferior and medial occipital lobe (Catani et al., 2002). Importantly, in the frontal lobe, the IFOF shares the territories with the uncinate fasciculus (UF). The UF connects the medial and lateral OFC, as well as the anterior temporal lobe, and is known to play an important role in regulating emotional responses and attaching emotion to visual information (Catani et al., 2003, Schmahmann et al., 2008). The involvement of OFC in the limbic system, together with the amygdala, cingulate cortex, and hippocampus, is very important. The OFC connects with the amygdala, which plays an important role in the interpretation of sensory, affective, and motivational information to produce reward outcomes (Rolls, 2019). The anterior cingulate cortex receives inputs from the OFC and amygdala with inputs from ventral stream, while the posterior cingulate cortex receives from dorsal stream areas including the parietal cortex and has connections with the hippocampal memory system (Rolls, 2019). The impairment of the OFC is consistently reported to be associated with apathy (Le Heron et al., 2018, Theleritis et al., 2014). Notably, a recent PET and MRI study in patients with AD found elevated ^11^C-PBB3 standardized uptake value ratios (a PET marker indicative of tau accumulation) in OFC, decreased OFC thickness and decreased FA in the UF to be significantly associated with increased scores of the Apathy Scale scores. Moreover, the path analysis indicated that increased ^11^C-PBB3 ratios in OFC affected apathy directly and through reduction of OFC thickness and subsequent decrease of FA in the UF (Kitamura et al., 2018). A number of MRI studies have also revealed the involvement of temporal and parietal cortex associated with apathy, both in AD (Balthazar et al., 2014) and MCI (Donovan et al., 2014, Guercio et al., 2015, Munro et al., 2015). Another study from our group with the same sample also found lower choline and myo-inositol in temporo-parietal cortex associated with apathy (Tumati et al., 2018). Indeed, the involvement of frontal and temporal gray matter and WM structures has been suggested to play an important role in regulation of goal-directed behavior (Brown and Pluck, 2000). Thus, alterations in the WM connecting these structures may be associated with apathy severity. Additionally, our findings are in line with a previous TBSS study in AD, which reported FA changes in the UF and right SLF associated with apathy (Hahn et al., 2013).

### 4.3 Projection pathways and apathy

In addition to the pathways discussed above, our study revealed that severity of apathy is inversely associated with decreased FA in bilateral anterior and superior corona radiata (ACR and SCR) and anterior thalamic radiation (ATR). A trend association also shown in corticospinal tract/internal capsule (IC). Projection fibers interconnect the cortex and subcortical structures (i.e. deep cerebral nuclei, brainstem nuclei, spinal cord), mostly projecting through the corona radiata, internal capsule, cerebral peduncles and brainstem (Catani et al., 2013). The projection fibers contain thalamocortical (ascending) fibers that project from subcortical nuclei (mainly thalamus) and terminate in the cortex, as well as motor corticofugal (descending) fibers, which run in opposite directions. The IC and corona radiata comprise of ascending fibers mainly from thalamus and descending fibers from fronto-parietal cortex to subcortical nuclei, including basal ganglia, brainstem nuclei and spinal cord. This complex projection conveys sensorial information to the cortex and transmits information necessary for the control of movement (Catani et al., 2013).

The anterior corona radiata (ACR) connects the anterior cingulate cortex to the striatum and other regions involved in behavior regulation (McCandliss, 2012), and is known as part of the limbic-thalamo-cortical loops (Wakana et al., 2004). This important loop has been proposed to underlie subtypes of apathy (Levy and Dubois, 2006), comprising different structures including the frontal lobe, thalamus, striatum, globus pallidus, limbic system and the connecting fibers. Noting that the frontal part of corona radiata (SCR and ACR) connects the PFC to the striatum and thalamus, and is part of the frontal-subcortical loops, we suggest that the aberrant connections of these fibers may result in attention deficit, emotional, and cognitive disorders, and may consequently also lead to increasingly severe apathy. The association between alterations in the ACR and the severity of apathy is supported by findings of previous studies in PD patients (Zhang et al., 2018), post stroke (Yang et al., 2015), and people with HIV infection (Kamat et al., 2014).

The anterior thalamic radiation (ATR) connects the mediodorsal and anterior thalamic nuclei to the prefrontal cortex and the anterior cingulate cortex. The thalamus is a complex structure composed of several nuclei, each interconnected with different cortical areas, located in diencephalon that operates as a relay station for most sensory and motor pathways and acts as a source of information for the frontal and motor areas (Catani et al., 2013). The anterior cingulate cortex plays an important role in regulating intention, monitoring and motivation, whilst the PFC is an important region in the cortico-subcortical network. Thus, in line with a previous study (Torso et al., 2015), we suggest that the disconnection of these fibers correlates with apathy severity.

Taken together, we propose that the more communication between frontal-limbic structures is disrupted, the higher severity of apathy is likely to be. More specifically, alterations of anterior cingulate cortex, orbital frontal cortex, and medial thalamus, as well as the disruption in the interconnections between these structures, may crucially underlie apathy (Kos et al., 2016, Le Heron et al., 2018).

A number of limitations of our study need to be considered. Firstly, we would like to note here that our data was collected with a well distributed range of apathy scores (Histogram in Supplementary Fig. 1). Thus, our data was suitable for correlation analysis. Importantly, however, there were only 8 patients clinically diagnosed with apathy, thus, we could not conduct subgroup analyses and differentiate an aMCI group with and without apathy. Therefore, our conclusions are limited to the correlation with apathy symptoms and not necessarily specific to clinically diagnosed apathy. However, as subjects with apathy are difficult to recruit, these results are valuable and provide insight into its neural correlates. Secondly, although the findings showed aberrant WM integrity in widespread areas to be associated with apathy, we were not able to capture the different dimensions of apathy. Future investigations using other multidimensional apathy measurements such as the Dimensional Apathy Scale (Radakovic and Abrahams, 2014) might better capture the sub-types of apathy and its correlates. Thirdly, our study consisted of a cross-sectional analysis, representing a snapshot of individuals at certain aMCI stages. Since the characteristics of aMCI are likely to change over time, cross-sectional surveys may not provide a complete picture. Longitudinal studies are clearly needed to reveal how the relationship between WM integrity, apathy, and aMCI may develop over time. Fourthly, although MMSE has been used widely to assess global cognition it is not considered sensitive for detecting cognitive decline in MCI. A recent systematic review showed that Montreal Cognitive Assessment (MoCA) is better in discriminating subjects with MCI and cognitively healthy individuals (Pinto et al., 2019). Lastly, we did not assess the presence of white matter lesions which are seen as white matter hyperintensities (WMH) on the brain scan as it requires a different MRI sequence (i.e. T2 weighted MRI). A previous study demonstrated that subjects having WMH were more likely to have elevated MD and reduced FA in general compared to healthy subjects not having WMH (Lange et al., 2014).

## 5 Conclusions

In conclusion, we found an association of apathy severity with widespread aberrations of WM integrity in aMCI. Our results suggest that the severity of apathy is associated with the disruption of a complex network of brain regions, not limited to frontal-subcortical circuits.

## Funding

This work was funded by the University Medical Center Groningen, Groningen, The Netherlands.

## CRediT authorship contribution statement

**Tania M. Setiadi:** Conceptualization, Formal analysis, Methodology, Visualization, Writing - original draft. **Sander Martens:** Conceptualization, Funding acquisition, Supervision, Writing - review & editing. **Esther M. Opmeer:** Investigation, Supervision, Writing - review & editing. **Jan-Bernard C. Marsman:** Data curation, Formal analysis, Writing - review & editing. **Shankar Tumati:** Conceptualization, Funding acquisition, Investigation, Writing - review & editing. **Fransje E. Reesink:** Investigation, Writing - review & editing. **Peter P. De Deyn:** Conceptualization, Investigation, Writing - review & editing. **André Aleman:** Conceptualization, Supervision, Writing - review & editing. **Branislava Ćurčić-Blake:** Conceptualization, Funding acquisition, Methodology, Supervision, Writing - review & editing.

## Declaration of Competing Interest

The authors declare that they have no known competing financial interests or personal relationships that could have appeared to influence the work reported in this paper.

## References

[b0005] Acosta-Cabronero J., Williams G.B., Pengas G., Nestor P.J. (2010). Absolute diffusivities define the landscape of white matter degeneration in Alzheimer’s disease. Brain.

[b0010] Adams K.B., Matto H.C., Sanders S. (2004). Confirmatory factor analysis of the geriatric depression scale. The Gerontologist.

[b0015] Andersson J.L.R., Skare S., Ashburner J. (2003). How to correct susceptibility distortions in spin-echo echo-planar images: application to diffusion tensor imaging. NeuroImage.

[b0020] Andersson J.L.R., Sotiropoulos S.N. (2016). An integrated approach to correction for off-resonance effects and subject movement in diffusion MR imaging. NeuroImage.

[b0025] Ardila A. (2008). On the evolutionary origins of executive functions. Brain Cogn..

[b0030] Ashburner J., Friston K.J. (2000). Voxel-based morphometry—the methods. NeuroImage.

[b0035] Balthazar M.L.F., Pereira F.R.S., Lopes T.M., da Silva E.L., Coan A.C., Campos B.M., Duncan N.W., Stella F., Northoff G., Damasceno B.P., Cendes F. (2014). Neuropsychiatric symptoms in Alzheimer's disease are related to functional connectivity alterations in the salience network: NPS in AD and Functional Connectivity Alterations of SN. Hum. Brain Mapp..

[b0040] Barbey A.K., Koenigs M., Grafman J. (2013). Dorsolateral prefrontal contributions to human working memory. Cortex.

[b0045] Basser P.J., Mattiello J., LeBihan D. (1994). MR diffusion tensor spectroscopy and imaging. Biophys. J ..

[b0050] Basser P.J., Mattiello J., Lebihan D. (1994). Estimation of the effective self-diffusion tensor from the NMR Spin Echo. J. Magn. Reson., Ser B.

[b0055] Beaulieu C. (2002). The basis of anisotropic water diffusion in the nervous system - a technical review. NMR Biomed..

[b0060] Brown R.G., Pluck G. (2000). Negative symptoms: the ‘pathology’ of motivation and goal-directed behaviour. Trends Neurosci..

[b0065] Cacciari C., Moraschi M., Di Paola M., Cherubini A., Orfei M.D., Giove F., Maraviglia B., Caltagirone C., Spalletta G. (2010). White matter microstructure and apathy level in amnestic mild cognitive impairment. J. Alzheimers. Dis..

[b0070] Carpenter P.A., Just M.A., Reichle E.D. (2000). Working memory and executive function: evidence from neuroimaging. Curr. Opin. Neurobiol..

[b0075] Catani M., Ffytche D.H. (2005). The rises and falls of disconnection syndromes. Brain.

[b0080] Catani M., Howard R.J., Pajevic S., Jones D.K. (2002). Virtual in vivo interactive dissection of white matter fasciculi in the human brain. NeuroImage.

[b0085] Catani M., Jones D.K., Donato R., Ffytche D.H. (2003). Occipito-temporal connections in the human brain. Brain.

[b0090] Catani M., Thiebaut de Schotten M., Catani M., Thiebaut de Schotten M. (2013). Atlas of Human Brain Connections (all tracts). Atlas of Human Brain Connections.

[b0095] Chase T.N. (2011). Apathy in neuropsychiatric disease: diagnosis, pathophysiology, and treatment. Neurotox. Res..

[b0100] Chilovi B.V., Conti M., Zanetti M., Mazzu I., Rozzini L., Padovani A. (2009). Differential impact of apathy and depression in the development of dementia in mild cognitive impairment patients. Dement. Geriatr. Cogn. Disord..

[b0105] Clark D.L., Boutros N.N., Mendez M.F. (2018). The brain and behavior: an introduction to behavioral neuroanatomy. The Brain and Behavior: An Introduction to Behavioral Neuroanatomy.

[b0110] Clarke D.E., Ko J.Y., Lyketsos C., Rebok G.W., Eaton W.W. (2010). Apathy and cognitive and functional decline in community-dwelling older adults: results from the Baltimore ECA longitudinal study. Int. Psychogeriatr..

[b0115] Clarke D.E., Reekum R.V., Simard M., Streiner D.L., Freedman M., Conn D. (2007). Apathy in dementia: an examination of the psychometric properties of the apathy evaluation scale. J. Neuropsychiatry Clin. Neurosci..

[b0120] Conde-Sala J.L., Turró-Garriga O., Piñán-Hernández S., Portellano-Ortiz C., Viñas-Diez V., Gascón-Bayarri J., Reñé-Ramírez R. (2016). Effects of anosognosia and neuropsychiatric symptoms on the quality of life of patients with Alzheimer's disease: a 24-month follow-up study: anosognosia and neuropsychiatric symptoms in AD. Int. J. Geriatr. Psychiatry.

[b0125] De Schotten M.T., Urbanski M., Duffau H., Volle E., Lévy R., Dubois B., Bartolomeo P. (2005). Neuroscience: direct evidence for a parietal-frontal pathway subserving spatial awareness in humans. Science (80-.).

[b0130] Donovan N.J., Wadsworth L.P., Lorius N., Locascio J.J., Rentz D.M., Johnson K.A., Sperling R.A., Marshall G.A. (2014). Regional cortical thinning predicts worsening apathy and hallucinations across the Alzheimer disease spectrum. Am. J. Geriatric Psychiatry.

[b0135] Fernandez-Martinez M., Molano A., Castro J., Zarranz J.J. (2010). Prevalence of neuropsychiatric symptoms in mild cognitive impairment and Alzheimer's disease, and its relationship with cognitive impairment.. Curr. Alzheimer Res..

[b0140] Folstein M.F., Folstein S.E., McHugh P.R. (1975). “Mini-mental state”. J. Psychiatr. Res..

[b0145] Gallagher D., Fischer C.E., Iaboni A. (2017). Neuropsychiatric symptoms in mild cognitive impairment: an update on prevalence, mechanisms, and clinical significance. Can. J. Psychiatry.

[b0150] Geda Y.E., Roberts R.O., Knopman D.S., Petersen R.C., Christianson T.J.H., Pankratz V.S., Smith G.E., Boeve B.F., Ivnik R.J., Tangalos E.G., Rocca W.A. (2008). Prevalence of neuropsychiatric symptoms in mild cognitive impairment and normal cognitive aging: population-based study. Arch. Gen. Psychiatry.

[b0155] Guercio B.J., Donovan N.J., Ward A., Schultz A., Lorius N., Amariglio R.E., Rentz D.M., Johnson K.A., Sperling R.A., Marshall G.A. (2015). Apathy is associated with lower inferior temporal cortical thickness in mild cognitive impairment and normal elderly individuals. J. Neuropsychiatry Clin. Neurosci..

[b0160] Haber S.N., Calzavara R. (2009). The cortico-basal ganglia integrative network: the role of the thalamus. Brain Res. Bull..

[b0165] Hahn C., Lim H.K., Won W.Y., Ahn K.J., Jung W.S., Lee C.U. (2013). Apathy and white matter integrity in Alzheimer’s disease: a whole brain analysis with tract-based spatial statistics. PLoS One.

[b0170] Hoare J., Fouche J.-P., Spottiswoode B., Joska J.A., Schoeman R., Stein D.J., Carey P.D. (2010). White matter correlates of apathy in HIV-positive subjects: a diffusion tensor imaging study. J. Neuropsychiatry Clin. Neurosci..

[b0175] Hofer S., Frahm J. (2006). Topography of the human corpus callosum revisited—comprehensive fiber tractography using diffusion tensor magnetic resonance imaging. NeuroImage.

[b0180] Hollerman J.R., Tremblay L., Schultz W. (1998). Influence of reward expectation on behavior-related neuronal activity in primate striatum. J. Neurophysiol..

[b0185] Hsu J.-L., Leemans A., Bai C.-H., Lee C.-H., Tsai Y.-F., Chiu H.-C., Chen W.-H. (2008). Gender differences and age-related white matter changes of the human brain: a diffusion tensor imaging study. NeuroImage.

[b0190] Inano S., Takao H., Hayashi N., Abe O., Ohtomo K. (2011). Effects of age and gender on white matter integrity. Am. J. Neuroradiol..

[b0195] Ismail Z., Smith E.E., Geda Y., Sultzer D., Brodaty H., Smith G., Agüera-Ortiz L., Sweet R., Miller D., Lyketsos C.G. (2016). Neuropsychiatric symptoms as early manifestations of emergent dementia: Provisional diagnostic criteria for mild behavioral impairment. Alzheimer's & Dementia.

[b0200] Jardri R., Pins D., Bubrovszky M., Despretz P., Pruvo J.-P., Steinling M., Thomas P. (2007). Self awareness and speech processing: An fMRI study. NeuroImage.

[b0205] Kamat R., Brown G.G., Bolden K., Fennema-Notestein C., Archibald S., Marcotte T.D., Letendre S.L., Ellis R.J., Woods S.P., Grant I., Heaton R.K. (2014). Apathy is associated with white matter abnormalities in anterior, medial brain regions in persons with HIV infection. J. Clin. Exp. Neuropsychol..

[b0210] Kazui H., Takahashi R., Yamamoto Y., Yoshiyama K., Kanemoto H., Suzuki Y., Sato S., Azuma S., Suehiro T., Shimosegawa E., Ishii K., Tanaka T., Nunomura A. (2016). Neural basis of apathy in patients with amnestic mild cognitive impairment. J. Alzheimers. Dis..

[b0215] Kim J.W., Lee D.Y., Choo IL.H., Seo E.H., Kim S.G., Park S.Y., Woo J.I. (2011). Microstructural alteration of the anterior cingulum is associated with apathy in Alzheimer disease. Am. J. Geriatric Psychiatry.

[b0220] Kitamura S., Shimada H., Niwa F., Endo H., Shinotoh H., Takahata K., Kubota M., Takado Y., Hirano S., Kimura Y., Zhang M.-R., Kuwabara S., Suhara T., Higuchi M. (2018). Tau-induced focal neurotoxicity and network disruption related to apathy in Alzheimer’s disease. J. Neurol. Neurosurg. Psychiatry.

[b0225] Kos C., van Tol M.-J., Marsman J.-B., Knegtering H., Aleman A. (2016). Neural correlates of apathy in patients with neurodegenerative disorders, acquired brain injury, and psychiatric disorders. Neurosci. Biobehav. Rev..

[b0230] Lanctôt K.L., Agüera-Ortiz L., Brodaty H., Francis P.T., Geda Y.E., Ismail Z., Marshall G.A., Mortby M.E., Onyike C.U., Padala P.R., Politis A.M., Rosenberg P.B., Siegel E., Sultzer D.L., Abraham E.H. (2017). Apathy associated with neurocognitive disorders: recent progress and future directions. Alzheimer's & Dementia.

[b0235] Lange R.T., Shewchuk J.R., Heran M.K.S., Rauscher A., Jarrett M., Brubacher J.R., Iverson G.L. (2014). To Exclude or not to exclude: further examination of the influence of white matter hyperintensities in diffusion tensor imaging research. J. Neurotrauma.

[b0240] Le Bihan D. (2003). Looking into the functional architecture of the brain with diffusion MRI. Nat. Rev. Neurosci..

[b0245] Le Heron C., Apps. M.A.J., Husain M. (2018). The anatomy of apathy: a neurocognitive framework for amotivated behaviour. Neuropsychologia.

[b0250] Levy R. (2012). Apathy: a pathology of goal-directed behaviour. A new concept of the clinic and pathophysiology of apathy. Revue Neurologique.

[b0255] Levy R., Dubois B. (2006). Apathy and the functional anatomy of the prefrontal cortex-basal ganglia circuits. Cereb. Cortex.

[b0260] Lezak M.D., Howieson D.B., Loring D.W., Hannay J.H., Fischer J.S. (2004). Neuropsychological Assessment.

[b0265] Liu J., Yin C., Xia S., Jia L., Guo Y., Zhao Z., Li X., Han Y., Jia J. (2013). White matter changes in patients with amnestic mild cognitive impairment detected by diffusion tensor imaging. PLoS One.

[b0270] Marin R.S. (1991). Apathy: a neuropsychiatric syndrome. J. Neuropsychiatry Clin. Neurosci..

[b0275] Marin R.S. (1990). Differential diagnosis and classification of apathy. Am. J. Psychiatry.

[b0280] Marin R.S., Biedrzycki R.C., Firinciogullari S. (1991). Reliability and validity of the apathy evaluation scale. Psychiatry Res..

[b0285] Marin R.S., Firinciogullari S., Biedrzycki R.C. (1993). The sources of convergence between measures of apathy and depression. J. Affect. Disord..

[b0290] Massimo L., Powers J.P., Evans L.K., McMillan C.T., Rascovsky K., Eslinger P., Ersek M., Irwin D.J., Grossman M. (2015). Apathy in frontotemporal degeneration: neuroanatomical evidence of impaired goal-directed behavior. Front. Hum. Neurosci..

[b0295] Matsumoto R., Nair D.R., LaPresto E., Najm I., Bingaman W., Shibasaki H., Lüders H.O. (2004). Functional connectivity in the human language system: a cortico-cortical evoked potential study. Brain.

[b0300] McCandliss B.D. (2012). Microstructural properties of white matter tracts are linked to the efficiency of specific attention networks. Cogn. Neurosci. Atten..

[b0305] Mori S., Wakana S., Zijl P.C.M.Van, Nagae-Poetscher L.M. (2005). MRI Atlas of Human White Matter.

[b0310] Mori S., Zhang J. (2006). Principles of diffusion tensor imaging and its applications to basic neuroscience research. Neuron.

[b0315] Munro C.E., Donovan N.J., Guercio B.J., Wigman S.E., Schultz A.P., Amariglio R.E., Rentz D.M., Johnson K.A., Sperling R.A., Marshall G.A. (2015). Neuropsychiatric symptoms and functional connectivity in mild cognitive impairment. J. Alzheimer’s Dis..

[b0320] Nijsten J.M.H., Leontjevas R., Pat-El R., Smalbrugge M., Koopmans R.T.C.M., Gerritsen D.L. (2017). Apathy: risk factor for mortality in nursing home patients. J. Am. Geriatr. Soc..

[b0325] Öngür D., Price J.L. (2000). The organization of networks within the orbital and medial prefrontal cortex of rats, monkeys and humans. Cereb. Cortex.

[b0330] Onyike C.U., Sheppard J.-M., Tschanz J.T., Norton M.C., Green R.C., Steinberg M., Welsh-Bohmer K.A., Breitner J.C., Lyketsos C.G. (2007). Epidemiology of apathy in older adults: the cache county study. Am. J. Geriatric Psychiatry.

[b0335] Ota M., Sato N., Nakata Y., Arima K., Uno M. (2012). Relationship between apathy and diffusion tensor imaging metrics of the brain in Alzheimer's disease: Relationship between apathy and DTI in AD. Int. J. Geriatr. Psychiatry.

[b0340] Palmer K., Di Iulio F., Varsi A.E., Gianni W., Sancesario G., Caltagirone C., Spalletta G. (2010). Neuropsychiatric predictors of progression from amnestic-mild cognitive impairment to Alzheimer's disease: the role of depression and apathy. J. Alzheimer’s Dis..

[b0345] Petersen R.C., Smith G.E., Waring S.C., Ivnik R.J., Tangalos E.G., Kokmen E. (1999). Mild cognitive impairment: clinical characterization and outcome. Arch. Neurol..

[b0350] Petersen R.C. (2004). Mild cognitive impairment as a diagnostic entity. J. Int. Med..

[b0355] Pinto T.C.C., Machado L., Bulgacov T.M., Rodrigues-Júnior A.L., Costa M.L.G., Ximenes R.C.C., Sougey E.B. (2019). Is the Montreal Cognitive Assessment (MoCA) screening superior to the Mini-Mental State Examination (MMSE) in the detection of mild cognitive impairment (MCI) and Alzheimer’s Disease (AD) in the elderly?. Int. Psychogeriatr..

[b0360] Radakovic R., Abrahams S. (2014). Developing a new apathy measurement scale: dimensional Apathy Scale. Psychiatry Res..

[b0365] Raimo S., Santangelo G., D’Iorio A., Trojano L., Grossi D. (2019). Neural correlates of apathy in patients with neurodegenerative disorders: an activation likelihood estimation (ALE) meta-analysis. Brain Imaging Behav..

[b0370] Richard E., Schmand B., Eikelenboom P., Yang S.C., Ligthart S.A., Moll van Charante E.P., van Gool W.A. (2012). Symptoms of apathy are associated with progression from mild cognitive impairment to Alzheimer’s disease in non-depressed subjects. Dement. Geriatr. Cogn. Disord..

[b0375] Robert P., Lanctôt K.L., Agüera-Ortiz L., Aalten P., Bremond F., Defrancesco M., Hanon C., David R., Dubois B., Dujardin K., Husain M., König A., Levy R., Mantua V., Meulien D., Miller D., Moebius H.J., Rasmussen J., Robert G., Ruthirakuhan M., Stella F., Yesavage J., Zeghari R., Manera V. (2018). Is it time to revise the diagnostic criteria for apathy in brain disorders? The 2018 international consensus group. Eur. Psychiatry.

[b0380] Robert P., Onyike C.U., Leentjens A.F.G., Dujardin K., Aalten P., Starkstein S., Verhey F.R.J., Yessavage J., Clement J.P., Drapier D., Bayle F., Benoit M., Boyer P., Lorca P.M., Thibaut F., Gauthier S., Grossberg G., Vellas B., Byrne J. (2009). Proposed diagnostic criteria for apathy in Alzheimer’s disease and other neuropsychiatric disorders. Eur. Psychiatry.

[b0385] Robert P.H., Berr C., Volteau M., Bertogliati C., Benoit M., Mahieux F., Legrain S., Dubois B. (2006). Neuropsychological performance in mild cognitive impairment with and without apathy. Dement. Geriatr. Cogn. Disord..

[b0390] Robinson H., Calamia M., Gläscher J., Bruss J., Tranel D. (2014). Neuroanatomical correlates of executive functions: a neuropsychological approach using the EXAMINER battery. J. Int. Neuropsychol. Soc..

[b0395] Rolls E.T. (2019). The cingulate cortex and limbic systems for emotion, action, and memory. Brain Struct. Funct..

[b0400] Schmahmann J.D., Smith E.E., Eichler F.S., Filley C.M. (2008). Cerebral white matter: neuroanatomy, clinical neurology, and neurobehavioral correlates. Ann. N. Y. Acad. Sci..

[b0405] Sherman C., Liu C.S., Herrmann N., Lanctôt K.L. (2018). Prevalence, neurobiology, and treatments for apathy in prodromal dementia. Int. Psychogeriatry.

[b0410] Smith S.M. (2002). Fast robust automated brain extraction. Hum. Brain Mapp..

[b0415] Smith S.M., Jenkinson M., Johansen-Berg H., Rueckert D., Nichols T.E., Mackay C.E., Watkins K.E., Ciccarelli O., Cader M.Z., Matthews P.M., Behrens T.E.J. (2006). Tract-based spatial statistics: voxelwise analysis of multi-subject diffusion data. NeuroImage.

[b0420] Smith S.M., Jenkinson M., Woolrich M.W., Beckmann C.F., Behrens T.E.J., Johansen-Berg H., Bannister P.R., De Luca M., Drobnjak I., Flitney D.E., Niazy R.K., Saunders J., Vickers J., Zhang Y., De Stefano N., Brady J.M., Matthews P.M. (2004). Advances in functional and structural MR image analysis and implementation as FSL. NeuroImage.

[b0425] Smith S., Nichols T. (2009). Threshold-free cluster enhancement: addressing problems of smoothing, threshold dependence and localisation in cluster inference. NeuroImage.

[b0430] Spalletta G., Fagioli S., Caltagirone C., Piras F. (2013). Brain microstructure of subclinical apathy phenomenology in healthy individuals: Apathy Phenomenology in Healthy Individuals. Hum. Brain Mapp..

[b0435] Starkstein S.E., Mizrahi R., Capizzano A.A., Acion L., Brockman S., Power B.D. (2009). Neuroimaging correlates of apathy and depression in Alzheimer's disease. J. Neuropsychiatry.

[b0440] Stella F., Radanovic M., Aprahamian I., Canineu P.R., de Andrade L.P., Forlenza O.V. (2014). Neurobiological correlates of Apathy in Alzheimer's Disease and mild cognitive impairment: a critical review. J. Alzheimer’s Dis..

[b0445] Stuss D.T. (2011). Functions of the frontal lobes: relation to executive functions. J. Int. Neuropsychol. Soc..

[b0450] Tagariello P., Girardi P., Amore M. (2009). Depression and apathy in dementia: same syndrome or different constructs? A critical review. Arch. Gerontol. Geriatr..

[b0455] Theleritis C., Politis A., Siarkos K., Lyketsos C.G. (2014). A review of neuroimaging findings of apathy in Alzheimer's disease. Int. Psychogeriatr..

[b0460] Tighe S.K., Oishi K., Mori S., Smith G.S., Albert M., Lyketsos C.G., Mielke M.M. (2012). Diffusion tensor imaging of neuropsychiatric symptoms in mild cognitive impairment and Alzheimer’s Dementia. J. Neuropsychiatry Clin. Neurosci..

[b0465] Torso M., Serra L., Giulietti G., Spanò B., Tuzzi E., Koch G., Caltagirone C., Cercignani M., Bozzali M. (2015). Strategic lesions in the anterior thalamic radiation and apathy in early Alzheimer’s disease. PLoS One.

[b0470] Tournier J.-D., Mori S., Leemans A. (2011). Diffusion tensor imaging and beyond: diffusion tensor imaging and beyond. Magn. Reson. Med..

[b0475] Tumati S., Opmeer E.M., Marsman J.B.C., Martens S., Reesink F.E., De Deyn P.P., Aleman A. (2018). Lower choline and myo-inositol in temporo-parietal cortex is associated with apathy in amnestic MCI. Front. Aging Neurosci..

[b0480] van Dalen J.W., van Wanrooij L.L., Moll van Charante E.P., Brayne C., van Gool W.A., Richard E. (2018). Association of Apathy with risk of incident dementia: a systematic review and meta-analysis. JAMA Psychiatry.

[b0485] Verhage, F., 1964. Intelligence and Age: Research on Dutch People aged Twelve to Seventy-seven Years Old. [In Dutch: Intelligentie en leeftijd. Onderzoek bij Nederlanders van twaalf tot zevenenzeventig jaar], Assen: Van Gorcum.

[b0490] Wakana S., Jiang H., Nagae-Poetscher L.M., van Zijl P.C.M., Mori S. (2004). Fiber tract–based atlas of human white matter anatomy. Radiology.

[b0495] Wen M.-C., Steffens D.C., Chen M.-K., Zainal N.H. (2014). Diffusion tensor imaging studies in late-life depression: systematic review and meta-analysis: meta-analysis in late-life depression. Int J Geriatr Psychiatry.

[b0500] Winkler A.M., Ridgway G.R., Webster M.A., Smith S.M., Nichols T.E. (2014). Permutation inference for the general linear model. NeuroImage.

[b0505] Yang S.R., Shang X.Y., Tao J., Liu J.Y., Hua P. (2015). Voxel-based analysis of fractional anisotropy in post-stroke apathy. PLoS One.

[b0510] Yesavage J.A., Brink T.L., Rose T.L., Lum O., Huang V., Adey M., Leirer V.O. (1982). Development and validation of a geriatric depression screening scale: a preliminary report. J. Psychiatr. Res..

[b0515] Zhang B., Xu Y., Zhu B., Kantarci K. (2014). The role of diffusion tensor imaging in detecting microstructural changes in prodromal Alzheimer's disease. CNS Neurosci. Ther..

[b0520] Zhang Y., Wu J., Wu W., Liu R., Pang L., Guan D., Xu Y. (2018). Reduction of white matter integrity correlates with apathy in Parkinson's disease. Int. J. Neurosci..

[b0525] Zhuang L., Sachdev P.S., Trollor J.N., Reppermund S., Kochan N.A., Brodaty H., Wen W. (2013). Microstructural white matter changes, not hippocampal atrophy, detect early amnestic mild cognitive impairment. PLoS One.

