# Supplementary material for: Widespread white matter aberration is associated with the severity of apathy in amnestic Mild Cognitive Impairment: Tract-based spatial statistics analysis
Source: Neuroimage Clin. 2021 Jan 19;29:102567. doi: 10.1016/j.nicl.2021.102567 (PMC7856325; doi:10.1016/j.nicl.2021.102567)

# Appendix A. Supplementary Materials

**Supplementary Table 1. AES-C sub-component characteristics.**

|  | **aMCI Group (n=29)** | | **Control Group (n=20)** | | | | **Group comparison**^a^ **(*p*)** |
| --- | --- | --- | --- | --- | --- | --- | --- |
|  | **Mean (*SD*)** | **Range** | **Mean (*SD*)** | | **Range** | |  |
| **Cognitive apathy** | 14.6 (4.9) | 8-24 | 13 (2.6) | | 9-18 | | U= 246 ( .37) |
| **Behavior apathy** | 8.31(3.3) | 5-15 | 6.6 (1.7) | | 5-10 | | U= 214 ( .11) |
| **Emotional apathy** | 3.6 (1.6) | 2-7 | 2.8 (1.0) | | 2-5 | | U= 216 ( .11) |
| **Others** | 3.6 (1.6) | 3-9 | | 3.8 (7.7) | | 3-5 | U=224 ( .16) |

^a^Group comparisons were performed with Mann-Whitney U-test.

**Supplementary Table 2. Brain regions showing lower fractional anisotropy (FA) associated with higher AES-C scores.**

| MNI coordinates^a^ | | | *p* value^b^ | | | Anatomical region | | |
| --- | --- | --- | --- | --- | --- | --- | --- | --- |
|  |  |  | *p*< .05 | | *p*< .08 |  |  |  |
| x | y | z | aMCI group (n=29) | Combined group (n=49) | | JHU DTI-81 WM label^c^ | JHU Tractography atlas^d^ |  |
| 20 | 25 | 25 | .04 | .06 | | R. ACR | - |  |
| -18 | 25 | 25 | .04 | .06 | | L. ACR | - |  |
| 18 | 2 | 38 | .03 | .06 | | R. SCR | - |  |
| -18 | -4 | 38 | .04 | .06 | | L. SCR | - |  |
| -14 | 26 | 19 | .04 | .07 | | L. Genu CC | - |  |
| 18 | 8 | 33 | .03 | .06 | | R. Body CC | - |  |
| -17 | 9 | 33 | .04 | .07 | | L. Body CC | - |  |
| -29 | 35 | 5 | .04 | .06 | | - | 34% L. IFOF/ 17% UF/ 11% ATR |  |
| 34 | -43 | 33 | .049 | .076 | | - | 21% R. SLF/ 3%SLF temporal part |  |
| 24 | 25 | 28 | .03 | .07 | | - | 3% R. ATR |  |
| -32 | 25 | 19 | .046 | .07 | | - | 3% L. ATR |  |

^a^Montreal Neurological Institute coordinates (MNI); ^b^Threshold-free cluster enhancement corrected *p* value; ^c^(Mori et al., 2005); ^d^(Wakana et al., 2004); Right; L, Left; ACR, Anterior Corona Radiata; SCR, Superior Corona Radiata; CC, Corpus Callosum; IFOF, Inferior Fronto-occipital Fasciculus; UF, Uncinate Fasciculus; ATR, Anterior Thalamic Radiation; SLF, Superior Longitudinal Fasciculus. Ant, anterior; post, posterior.

**Supplementary Table 3. Brain regions showing a trend association between lower fractional anisotropy (FA) and higher AES-C scores in aMCI patients.**

| MNI coordinates^a^ | | | *p*< .065 | | Anatomical region | | |
| --- | --- | --- | --- | --- | --- | --- | --- |
|  |  |  |  |  |  | | |
| x | y | z | aMCI group (n=29) | | JHU DTI-81 WM label^c^ | JHU Tractography atlas^d^ | Atlas of Human Brain Connections^e^ |
| 19 | -35 | 38 | .06 | R. PCR | |  |  |
| 15 | 46 | 27 | .053 | - | | 18% R. Forceps minor |  |
| -19 | 38 | 23 | .06 | - | | 29% L. Forceps minor/ 8% ATR |  |
| 14 | 3 | 56 | .06 | - | | - | R. CST/IC |

^a^Montreal Neurological Institute coordinates (MNI); ^b^Threshold-free cluster enhancement corrected *p* value; ^c^(Mori et al., 2005); ^d^(Wakana et al., 2004); ^e^(Catani et al., 2013); R, Right; L, Left; PCR, Posterior Corona Radiata; ATR, Anterior Thalamic Radiation; CST, Corticospinal Tracts; IC, Internal Capsule.

**Supplementary Figure 1. AES-C histogram in aMCI patients.**

#
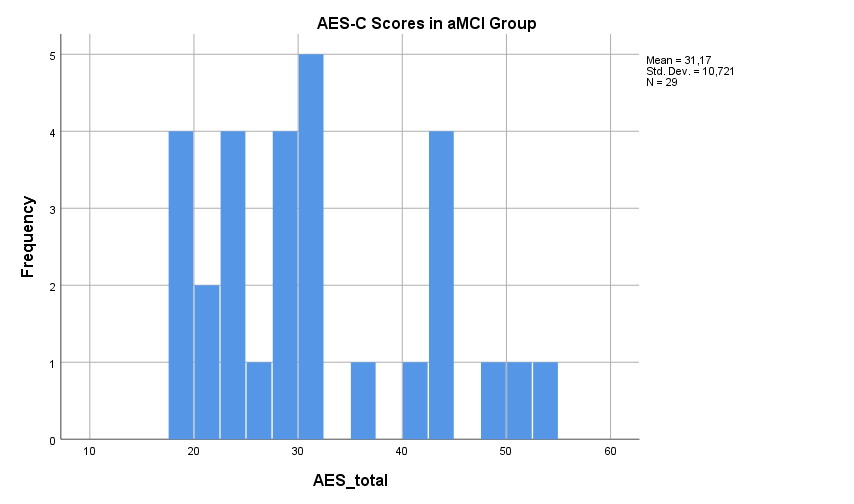

Supplement: Supplementary data 1 [file mmc1.docx]
